# Co-Registration of *ex vivo* Surgical Histopathology and *in vivo* T2 weighted MRI of the Prostate via multi-scale spectral embedding representation

**DOI:** 10.1038/s41598-017-08969-w

**Published:** 2017-08-18

**Authors:** Lin Li, Shivani Pahwa, Gregory Penzias, Mirabela Rusu, Jay Gollamudi, Satish Viswanath, Anant Madabhushi

**Affiliations:** 1 Department of Biomedical Engineering, Case Western Reserve University, Cleveland, Ohio 44106 United States of America; 2 Department of Radiology, Case Western Reserve University, Cleveland, Ohio 44106 United States of America; 3 GE Global Research, Niskayuna, New York 12309 United States of America; 4 University Hospitals, Cleveland, Ohio 44106 United States of America

## Abstract

Multi-modal image co-registration via optimizing mutual information (MI) is based on the assumption that intensity distributions of multi-modal images follow a consistent relationship. However, images with a substantial difference in appearance violate this assumption, thus MI directly based on image intensity alone may be inadequate to drive similarity based co-registration. To address this issue, we introduce a novel approach for multi-modal co-registration called Multi-scale Spectral Embedding Registration (MSERg). MSERg involves the construction of multi-scale spectral embedding (SE) representations from multimodal images via texture feature extraction, scale selection, independent component analysis (ICA) and SE to create orthogonal representations that decrease the dissimilarity between the fixed and moving images to facilitate better co-registration. To validate the MSERg method, we aligned 45 pairs of *in vivo* prostate MRI and corresponding *ex vivo* histopathology images. The dataset was split into a learning set and a testing set. In the learning set, length scales of 5 × 5, 7 × 7 and 17 × 17 were selected. In the independent testing set, we compared MSERg with intensity-based registration, multi-attribute combined mutual information (MACMI) registration and scale-invariant feature transform (SIFT) flow registration. Our results suggest that multi-scale SE representations generated by MSERg are found to be more appropriate for radiology-pathology co-registration.

## Introduction

**Figure 1:**
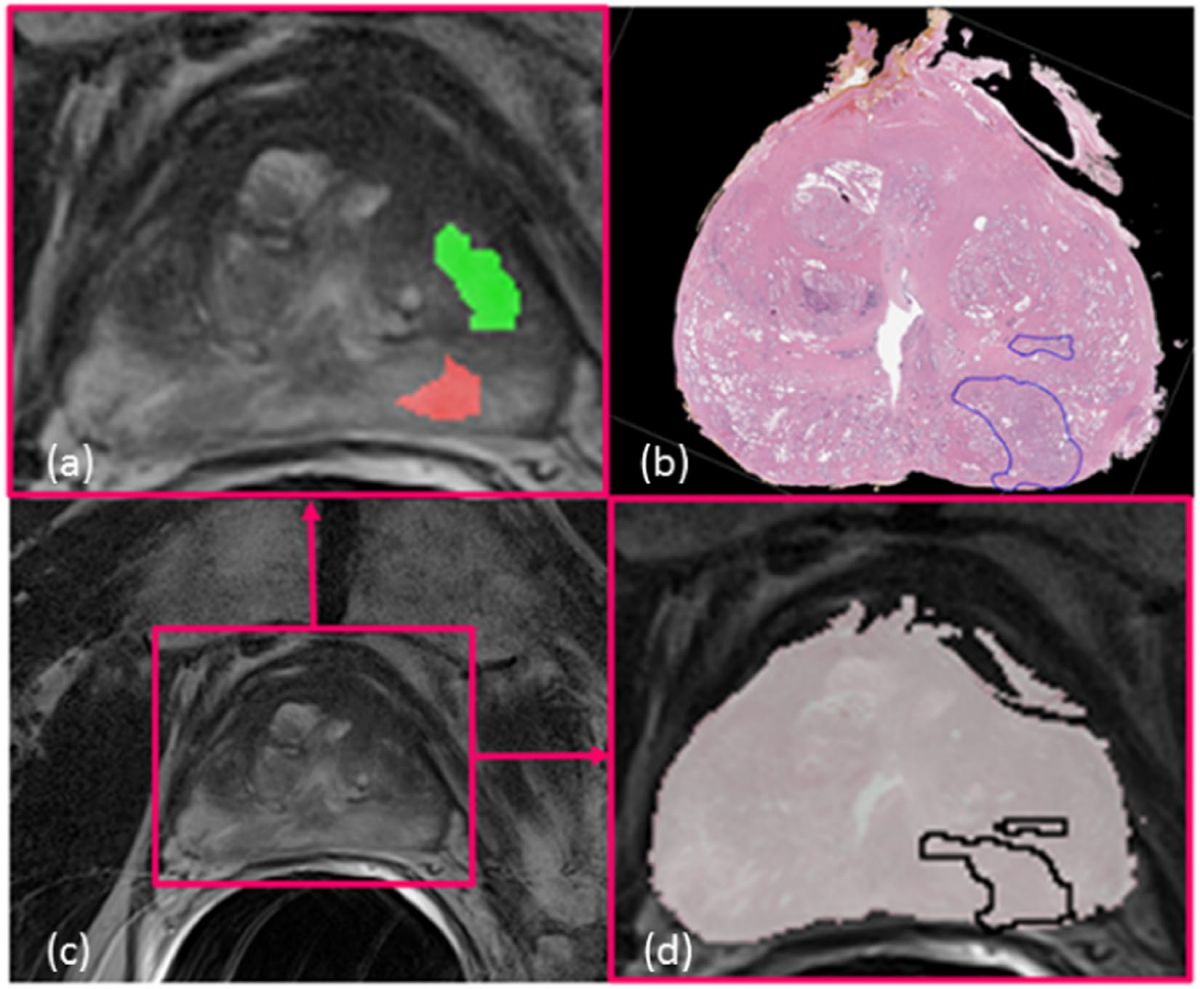
Illustration of a pair of corresponding *in vivo* prostate MRI (**c**) and *ex vivo* histology images (**b**). On panel **(a)** one can observe the cancer annotations made by two radiologists (green and red) unblinded to the corresponding histology images. The divergent annotations made by the two radiology readers, in spite of having access to the pathology images, suggests the need for accurate co-registration of pathology and radiology imaging in order to ascertain the ground truth extent for disease on radiology imaging ((**d**) the overlap registration visualization).

In spite of different radiographic imaging modalities (e.g. MRI, Ultrasound) being available for prostate cancer diagnosis, a definitive ascertainment of disease extent is only possible by histopathologic examination on surgically excised specimens^1^. Diagnosis and delineation of prostate cancer regions based on routine MRI may suffer from inter-rater differences. Figure 1 illustrates the challenges of relying solely on radiology readers to determine ground truth for disease extent using imaging alone.

Consequently, there has been a recent appreciation of the need to identify alternative image representations that can complement intensity information which includes image gradients^2^, co-occurrence information^3^, and image segmentations^4^ for the purposes of multi-modal image co-registration. Texture features, e.g. Gabor wavelets^5^ provide multi-scale, multi-oriented textured representations of the original images^6^. The intuition behind the texture feature representation is that similar underlying structural attributes (e.g. edge patterns) can be identified in the two images to be registered. Texture features extracted at differently sized neighborhoods enable the capturing of different underlying structural cues^6, 7^. In addition, certain scales might result in expression of cues or features that are not expressed at other scales. Hence, it becomes critical to explicitly consider the scales at which the features are extracted and develop better representations in a scale-specific manner. The challenge though is identifying which representations are most appropriate for multi-modal co-registration. Previous registration methods^6–10^ involving texture analysis did not explicitly interrogate the role of texture as a function of feature scale. MSERg aims to leverage the use of multiple feature scales for creating different image representations, thereby increasing the similarity of the multi-modal images and thus co-registration accuracy.

Though there are a number of extant texture features (e.g. Haralick^11^, Laws^12^, Gabor^5^), there also exists substantial redundancy between these features. The use of dimensional reduction (DR) methods to project high dimensional features into lower dimensional spaces allows for the construction of new transformed representations that can robustly drive multi-modal co-registration. Independent component analysis (ICA) is a method for decomposing mixture data into lower dimensional orthogonal features, with the most salient information obtained by minimizing mutual information (MI) and maximizing non-Gaussianity within the data^13^. Li *et al*.^6^ showed that using ICA could reduce redundancy between multi-channel image texture features, in turn improving the co-registration result. ICA does not, however, implicitly rank the order of the independent components (ICs) according to their importance.

Spectral embedding (SE) is a nonlinear DR method for projecting data into a low-dimensional manifold from a high-dimensional space^10^. This method takes the dissimilarity between two images as a weighted affinity matrix and applies eigenvalue decomposition to obtain the corresponding eigenvectors and eigenvalues. The approach orders the components that best preserve the structure of the high-dimensional manifold by selecting the eigenvectors corresponding to the minimum eigenvalues as the top components^10^. In addition, applying SE onto the textural ICs extracted at each scale can help preserve the nonlinear relationships between the target and template images and hence potentially facilitate more accurate co-registration.

MI is a popular similarity measure for multi-modal image co-registration where the goal is to try to maximize the information shared between two images^14^. MI mitigates the sensitivity of intensity-difference-based similarity measures on different image modalities by measuring intensity distribution of the images to be co-registered. Unfortunately, in spite of these advantages, MI is inadequate at robustly registering multi-modal images with substantially different appearance characteristics^1^. For example, the appearance of tissue and anatomical structures on prostate MRI and corresponding *ex vivo* histopathology are vastly different. (Figure 1) The shape of the excised prostatectomy specimen tends to substantially change during the process of tissue fixation, slicing and sectioning. The tissue preparation procedure has also been found to cause tissue loss and deformation. Similarly, the shape of *in vivo* MRI can undergo substantial deformation under pressure from surrounding organs such as the bladder. Furthermore, during MRI acquisition, the presence of an endorectal coil could also induce deformations in the natural shape of the prostate. Hence the goal of this work is to identify image representations which maximize the similarity between the fixed (MRI) and moving (histopathology) images. However, there is a need for higher dimensional similarity functions to be able to combine multiple different image representations to adequately co-register diverse looking images. To meet this need, we employ the *α*-MI, which shows great performance in high dimensional registration^15^, as the similarity measure.

## Previous Work and Brief Overview of Approach

As previously mentioned, there has been substantial interest in identifying alternative image representations for multi-modal co-registration. Chappelow *et al*.^8, 9^ presented a texture-feature-based registration method to improve multi-modal registration performance called multi-attribute combined mutual information (MACMI). The approach used a set of multiple image texture features to complement image intensity information including the first and second order statistical and gradient features. MACMI yielded a noticeable improvement in alignment accuracy between images of different modalities, evaluations having been performed on both synthetic and clinical studies. Multi-modal co-registration in high dimensional spaces is difficult to drive using traditional MI approximated by the ‘histogram-based plug-in’ estimation^16^. Thus, MACMI typically could not accommodate more than 2D or 3D spaces for co-registration^9^. Instead of depending on density estimation, Neemuchwala *et al*.^17^ computed the length of entropic graphs, such as the *k*-Nearest Neighbor graph (*k* NNG) and the minimal spanning tree (MST), to estimate graph entropic similarity measures. The graph entropic similarity measures, such as *α*-MI, can potentially eliminate the low dimensionality constraint of ‘histogram-based plug-in’ estimation^16^ during calculation of MI. Staring *et al*.^15^ applied entropy graphs to address the deformable registration of cervical MRI and solved the high dimensional registration problem by deriving the analytic derivative of *α*-MI with respect to the transformation parameters.

Furthermore, ICA has been employed to eliminate redundant representations from within a larger set of extracted textural features^6^. However, the disadvantage of ICA is its unordered output ICs, hence it is impossible to fix the alternative representations for the fixed and moving images and thus the registration performance will most likely not be robust in spite of the repeated application of ICA. Hence, we employ spectral embedding onto the ICA representations. This enables generation of a series of ordered spectral vectors which are arranged in order of the amount of variance captured in the ICA component space. Thus, by having a ranked set of vectors, we can employ the top spectral embedding vectors to drive the co-registration. In addition, ICA is a linear DR method. For the MRI and *ex vivo* pathology specimens that we are attempting to co-register, a linear embedding representation may not adequately capture the non-linear deformations induced in both the imaging and the pathology data during the acquisition process. Spectral embedding is a nonlinear DR method and hence the resulting vectors could potentially better capture the non-linear deformations induced in the imaging and pathology images.

**Figure 2:**
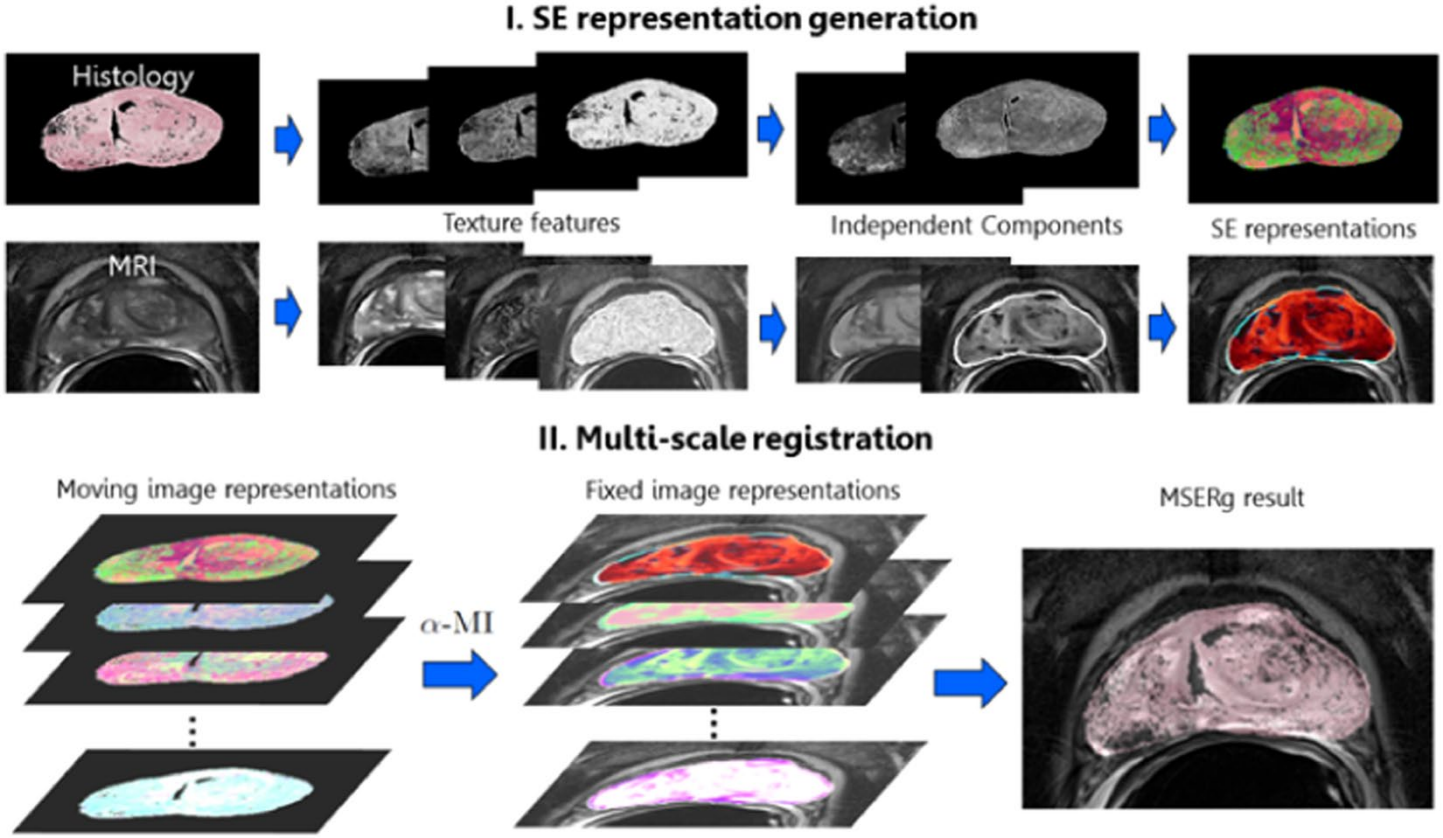
MSERg comprises 2 main modules: Firstly a SE representation is constructed at each scale. (1) Texture feature extraction is performed at each length scale within both the fixed and moving images. (2) ICA is then performed at each texture feature length scale, and (3) spectrally embed the ICs extracted from the texture features at each length scale. The second module involves multi-scale registration. This module comprises the following main steps. (1) Identifying and combining image representations at optimal length scales in order to drive co-registration and (2) using *α*-MI to combine the scale spaces of spectral embedding vectors in order to facilitate radiology-pathology co-registration.

In this paper, we present a new co-registration method, MSERg, that employs textural features, ICA, spectral embedding to identify the most representative feature scales and then combines these new multi-scale image representations via *α*-MI to facilitate more accurate multi-modal co-registration. Specifically, our approach explores the influence of scale length of extracted texture features on multi-modal co-registration. Figure 2 provides an illustrative rendering of the overall workflow comprising our method.

Co-registration performance was compared with intensity-based registration, a texture-feature-based registration, named as multi-attribute combined mutual information (MACMI) registration^18^, scale-invariant feature transform (SIFT) based registration, named as SIFT flow^19^, and single-scale Spectral embedding representation registration (SSERg). SIFT flow is a registration algorithm developed by Liu *et al*.^19^ and it employs the features extracted by SIFT descriptors to characterize the correspondence between the fixed and moving images. SIFT flow can be used to register images of great textural and appearance difference^19^. Our experiment comprised of clinical and synthetic datasets. In the clinical experiment, We applied the Dice similarity coefficient (DSC) to evaluate the alignment of prostate capsule boundaries between the histopathology images and corresponding MRI which segmented by radiologists. For local registration accuracy evaluation, DSC was employed to evaluate the registration accuracy by comparing the registration results against the tumor annotations mapped from histopathology images via manual registration. In addition, the root mean squared distance (RMSD) was used to measure the distance between the corresponding landmarks between the histopathology images and MRI in millimeters. In the synthetic experiments, We firstly applied synthetic non-linear B-splines deformations on the proton density (PD) images and then registered the corresponding T1 weighted and T2 weighted images in order to recover the original applied deformation. We then compared the recovered deformation against the induced deformation to evaluate the performance of the co-registration scheme. The mean deformation difference (MDD) was employed to quantify the recovered deformation errors compared to the induced deformation.

## Results

### Similarity improvement

**Figure 3:**
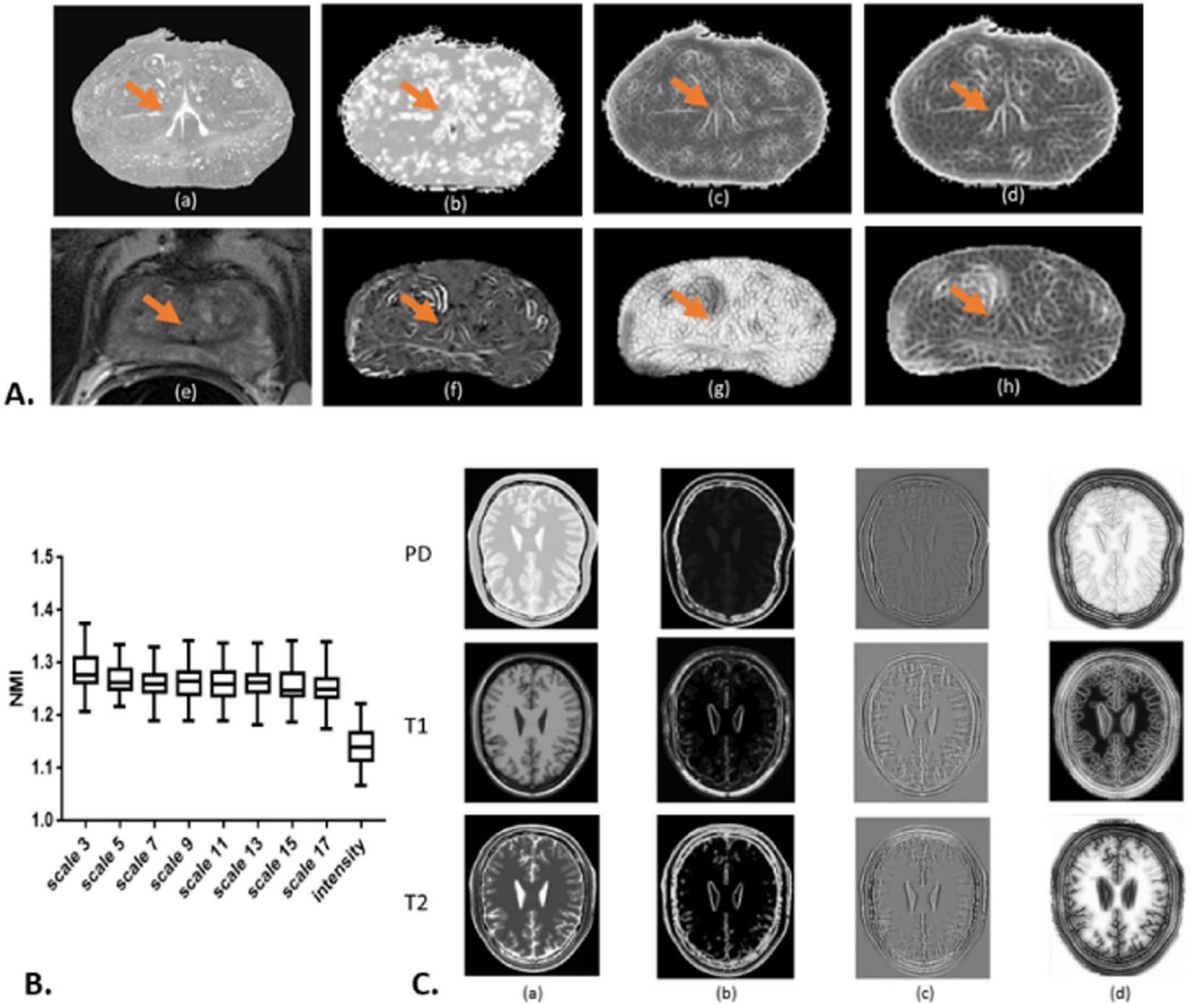
In Figure 3 Panel A, sub-panel (a) shows the down-sampled gray scale histopathology image and (e) shows the corresponding T2 weighted MRI slice. (b–d) are SE based representations of histopathology and (f–h) are MRI derived SE representations at scales *κ* ∈ {3, 11, 17}, respectively. The orange arrows point to the urethra structures. Panel B illustrates the NMI distribution for each scale representation across 45 pairs of histopathology images and MRI. Marked improvement in similarity is observed across all scales and reflected via a greater NMI of SE representations compared to intensity - based representations. In Panel C, column (a) shows the intensity representation of corresponding PD, T1 and T2 MRI and columns (b–d) show their corresponding SE representations at scales *κ* ∈ {3, 9, 17}, respectively. SE representations across different scales emphasize different attributes in the image, attributes that only become apparent at specific length scales. For instance, for clinical data, the region corresponding to the urethra is de-emphasized in the representations shown in (b) and (g). On the other hand, (c), (d), (f) and (h) highlight and emphasize the same regions. In addition, synthetic data SE representations in Panel C, sub-panel (c) illustrate underlying similarities across different modalities compared to sub-panels (a), (b) and (d).

Figure 3 panel B shows a box plot of NMI distribution among 45 pairs of MRI and histology images with original intensity representation and the SE representations with scale *κ* ∈ {3, 5, 7, 9, 11, 13, 15, 17}. Because each individual scale SE representation consists of 3 SE vectors, we use the average NMI value of these 3 corresponding SE vectors to represent the similarity level between SE representations of MRI and histology images. All the SE representations used in this experiment show greater NMI values than the intensity signal with moderate variance among different scales. The result indicates that SE representations could convert images of different modalities, MRI and histopathology images, to show more substantial similarity compared to the original intensity representation. In addition, SE representations of different scales extract different textural information. In Figure 3 panel A, (a) and (e) are the intensity representations of the moving and fixed images. (b–d) are histopathology SE representations and (f–h) are the corresponding MRI SE representations at scales *κ* ∈ {3, 11, 17}, respectively. The SE representation at scale *κ* = 17 shows more similar appearance between the two modalities compared to other image representations. The influence of length scales on morphologic cues within the images is clearly illustrated. For instance, the region corresponding to the urethra is de-emphasized in the representations and at the length scales shown in (b) and (g). On the other hand, (c), (d), (f), and (h) highlight and emphasize the same regions. Panel C demonstrates the intensity representations of brain PD, T1 weighted and T2 weighted MRI in column (a) and SE representations at scale *κ* ∈ {3, 9, 17} respectively in columns (b)-(d). Similar to the clinical data shown in panel A, SE representations at different scales extract divergent morphology and texture features. Synthetic data SE representations in panel C column (c) illustrate underlying similarities across different modalities compared respectively to columns (a), (b) and (d). These results reinforce our approach, that different morphological structures are emphasized or suppressed across different length scales and only a multi-scale representation can optimally help determine image similarity in order to aid in multimodal co-registration.

### Evaluation of MSERg with other representation-based registration methods

MSERg combined the scale *κ* ∈ {5, 7, 17} for clinical data and the scale *κ* ∈ {3, 5, 7} for synthetic data. These scale combinations were selected based on the evaluation of single-scale representation based registration performance on the independent learning set. For clinical data, the registration of the scale *κ* ∈ {3, 7, 17} show averagely better tumor annotation alignment than other scales. For synthetic data, MMD values between the induced deformation and recovered deformation of the scale *κ* ∈ {3, 5, 7} are less than other scales.

#### MSERg and Intensity-based Registration

**Figure 4:**
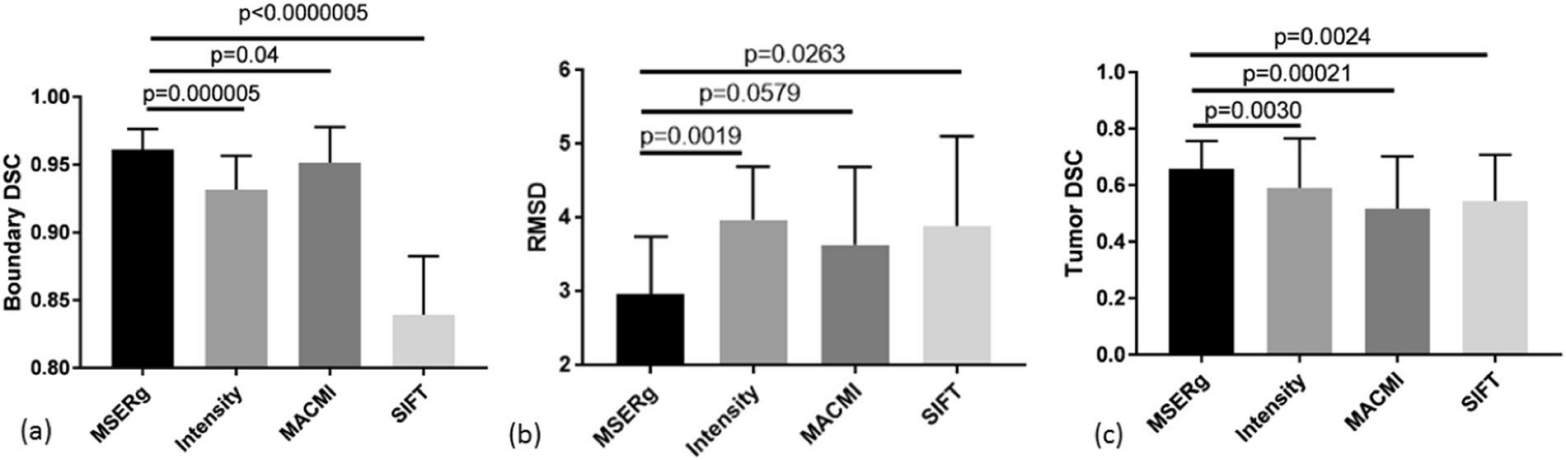
MSERg, MACMI, SIFT and Intensity-based Registration comparison in terms of prostate capsule boundary DSC, landmark RMSD and tumor DSC with statistical significance analysis testing on the clinical test set. Greater DSC value and lower RMSD indicate better annotation alignment and thus a better registration result. Statistical significance testing was performed between each pair of methods with respect to MSERg in terms of capsule boundary DSC, landmark RMSD and tumor DSC. In 8 of the 9 different comparisons performed, MSERg statistically significantly (p < 0.05) outperformed the other methods for the cases in the clinical test set.

For clinical data, MSERg illustrates a better across the board registration performance with less variance in the testing set compared with intensity-based registration. (Table 1) Furthermore, MSERg shows statistically significant performance improvement on all the three registration evaluation measurements. (Figure 4) Additionally, Figure 6 illustrates a clinical registration case showing intensity-based, MACMI, SIFT flow and MSERg registration results qualitatively. MSERg outperforms the other methods by showing the most accurate tumor ROI annotation, landmarks and capsule boundary alignment.

**Table 1:** Quantitative evaluation of representation-based registration on the clinical and synthetic testing sets.

Evaluation methods	Intensity-based Registration	MACMI	MSERg	SIFT
tumor lesion DSC	0.59 ± 0.17	0.52 ± 0.18	0.66 ± 0.10	0.54 ± 0.16
capsule boundary DSC	0.93 ± 0.02	0.95 ± 0.03	0.96 ± 0.01	0.84 ± 0.04
RSMD	3.97 ± 0.70	3.64 ± 1.02	2.96 ± 0.76	3.89 ± 1.17
MDD (T1 to PD)	33.78 ± 17.80	2.53 ± 1.05	1.45 ± 0.43	3.51 ± 0.73
MDD (T2 to PD)	41.89 ± 16.19	1.82 ± 1.00	0.50 ± 0.28	1.36 ± 0.62

(N = 25 for both datasets) The table shows the mean and the standard derivation of tumor DSC, prostate boundary DSC and landmark RMSD for clinical dataset and MDD for synthetic dataset. Note that the greater the DSC, the lower the RMSD and MDD indicate the more accurate result.

**Figure 5:**
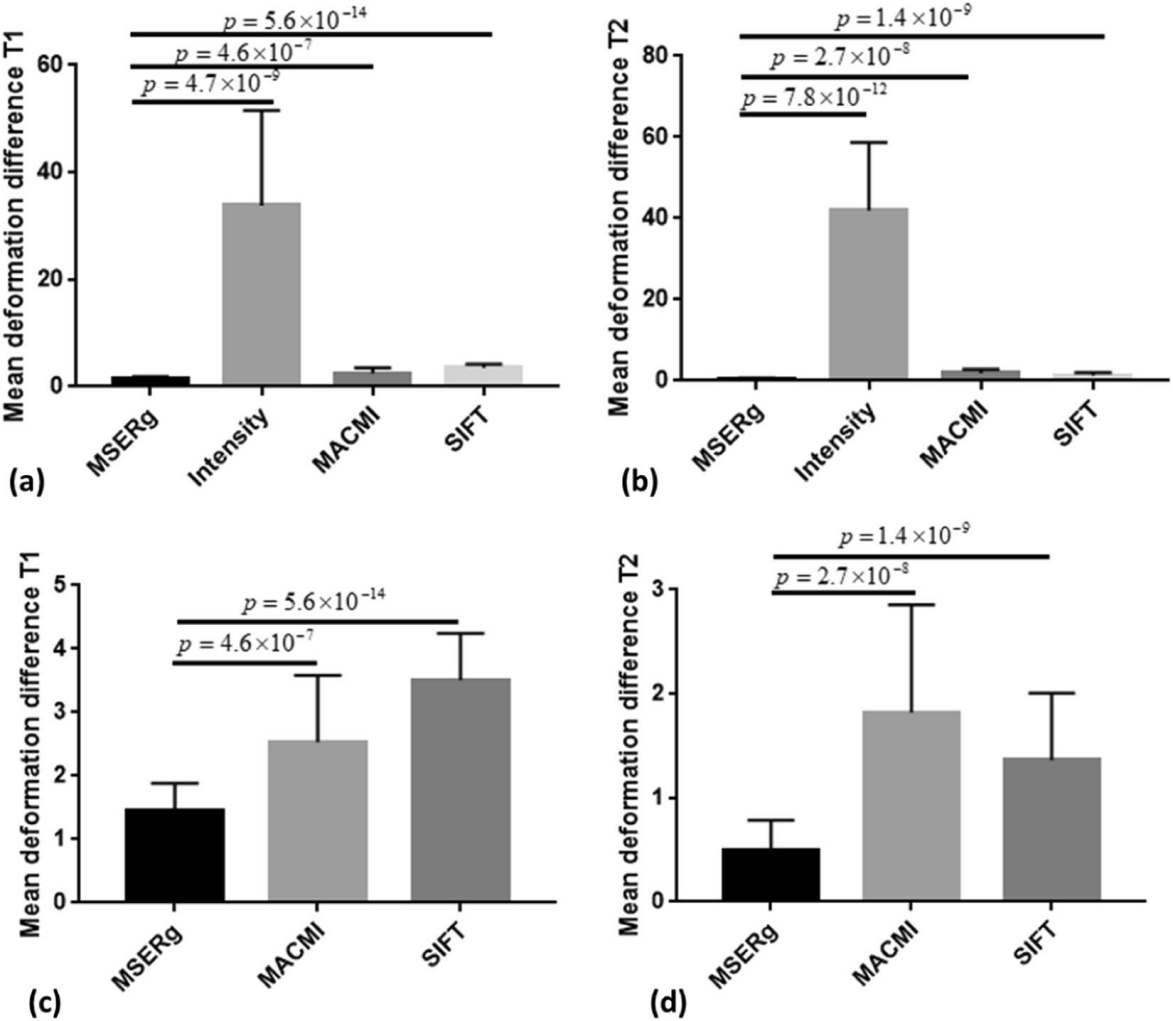
MSERg, MACMI, SIFT and Intensity-based Registration comparison in terms of MDD with statistical significance analysis on synthetic test set (25 cases of PD,T1 and T2 weighted MRI). Lower the MDD value indicates less difference between the recovered deformation and the induced deformation and thus a better registration result. (**a**) shows the registration results with PD images as fixed images and T1 weighted images as moving images and (**b**) with T2 weighted images as moving image. (**c**) and (**d**) exclude the intensity-based results to demonstrate comparison between MSERg, MACMI and SIFT methods more specifically. MSERg has the least MDD from the induced deformation compared with the other registration methods statistically significantly.

For synthetic data, the registration performance is evaluated via MDD. Table 1 and Fig. 5 demonstrate the quantitative performance evaluation among the four registration methods. MSERg has the least difference between the results and the induced deformation in both multimodal registration cases while intensity-based registration has the greatest difference.

#### MSERg and Texture-feature-based Registration

MACMI, a texture-feature-based-registration, although employs texture features like MSERg, the use of scale selection, ICA and SE appears confers a competitive advantage to MSERg. Figures 4 and 5 show the registration results evaluation with statistical significance analysis for clinical and synthetic dataset respectively. MSERg does not significantly outperform MACMI at a 95% confidence limit via landmark RMSD evaluation in clinical dataset. However, MSERg has significantly better boundary alignment and tumor annotation alignment accuracy.

#### MSERg and SIFT flow

**Figure 6:**
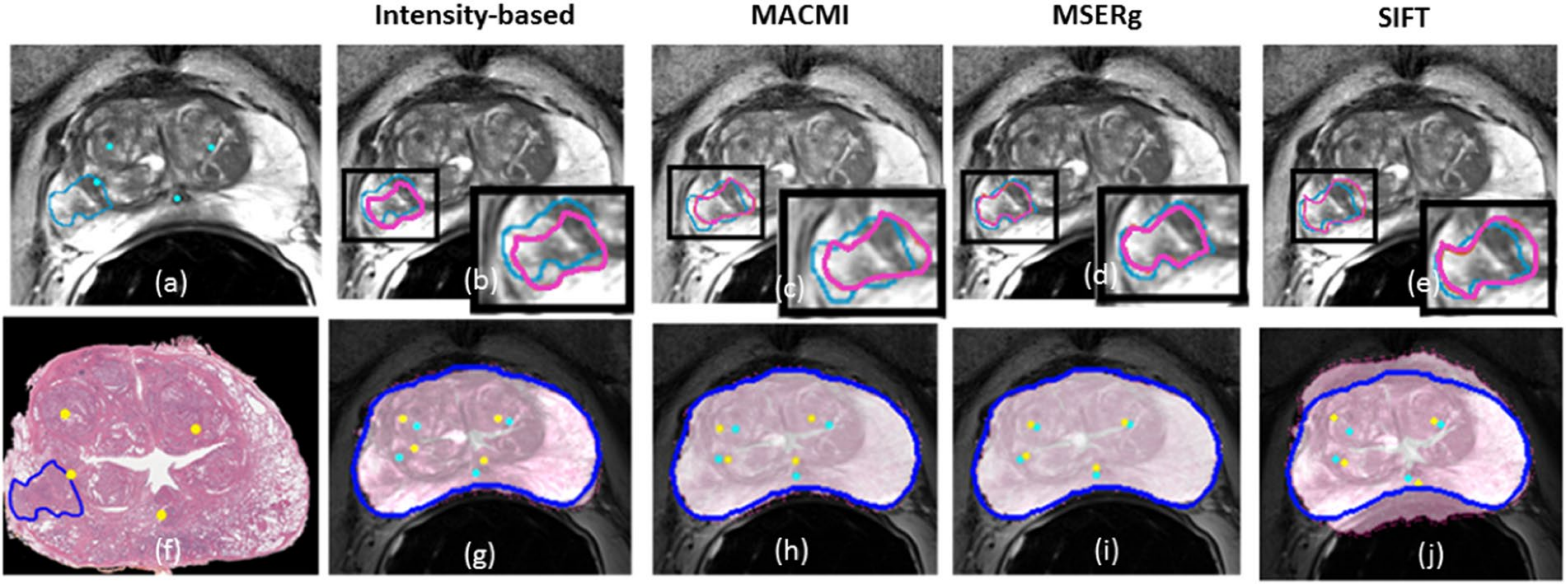
Illustration of a clinical registration case. (**a**), (**f**) are the fixed and moving images with landmarks annotated by the pathologist and the radiologist. The tumor ROI annotations on the pathology image are made by the pathologist and the corresponding tumor ROI on the MRI are mapped from pathology annotations obtained via manual registration. (**b**), (**g**) show the registration results of intensity-based registration; (**c**), (**h**) of MACMI, (**d**), (**i**) of MSERg and (**e**), (**j**) of SIFT, respectively. The light blue lines in panels (**b**–**e**) illustrate the tumor ROI on a T2-weighted MRI slice and the magenta lines highlight the corresponding ROI on the transformed pathology image. (**g**–**j**) illustrate the landmark alignment results where the cyan colored landmarks represent those identified on the MRI, while the yellow colored landmarks represent those corresponding locations on the transformed pathology image. The dark blue lines in (**g**–**j**) illustrate the capsule boundary on MRI.

SIFT is a descriptor to characterize image local intensity gradient information^20^. SIFT flow adopts SIFT descriptors that aligns images of very different texture and appearances based on local gradient information^19^. However, in case of *in vivo* T2 weighted MRI and *ex vivo* histology image registration, MSERg shows more accurate and robust results across 25 cases in the testing set. Especially, SIFT flow results have a poor boundary alignment accuracy compared with the other three registration methods. (Figures 4(a) and 6(j)) In addition, the MMD values of SIFT flow method in synthetic experiments are greater than MMD value of MSERg according to Figure 5. Thus, MSERg outperformed SIFT flow registration consistently on both clinical and synthetic datasets.

#### MSERg and SSERg

**Figure 7:**
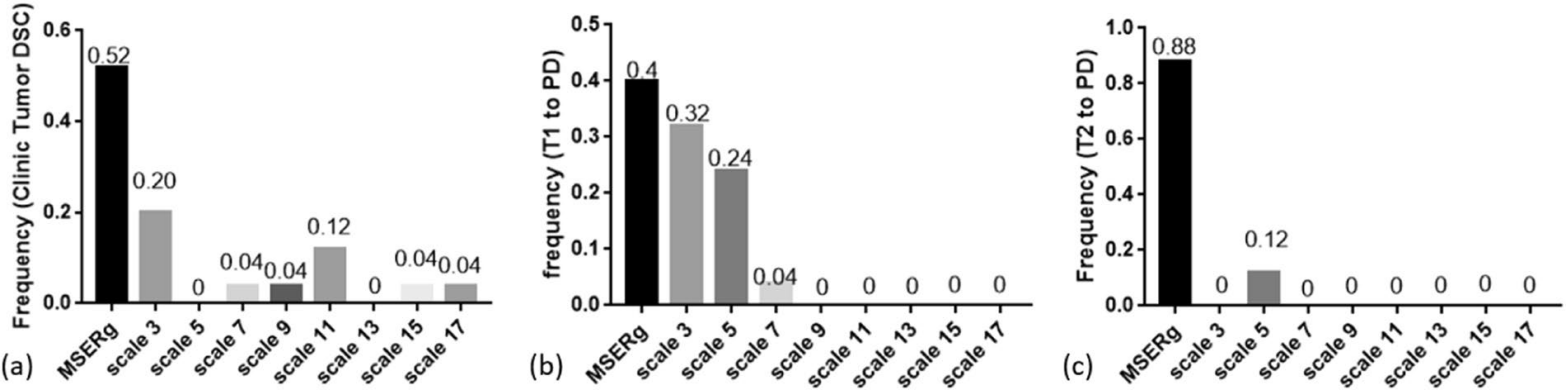
Illustration of a frequency plot showing in how many instances either SSERg or MSERg registration yielded the best registration result. We compared 8 different SSERg combinations along with MSERg on the same 25 clinical and synthetic testing cases. (**a**) Shows the frequency of each registration method having the greatest Tumor DSC within the clinical testing set. (**b**) and (**c**) illustrate the frequency of each registration method with the least mean deformation difference (MDD), in case of T1 to PD and T2 to PD registration respectively. Compared to SSERg, MSERg has more accurate and robust registration performance across different datasets.

Figure 7 illustrates a frequency plot showing in how many instances either the SSERg or MSERg registration yielded the best result among clinical (N = 25) and synthetic (N = 25) testing sets. Although MSERg cannot outperform SSERg with every individual scale for every given experiment or dataset, overall MSERg dominates as the best performing registration scheme the majority of the time. The results clearly suggest that integrating multiple length scales offers an advantage in terms of multi-modal co-registration, the majority of the time.

Furthermore, the same scale combination kept in both T1 to PD and T2 to PD registrations and MSERg more frequently shows the best registration performance in both multimodal registration datasets. However, the results in Figure 7 also suggest that there is little consistency in performance of a specific scale across different experiments. In other words, there is no “magic scale”. For example, though scale *κ* = 3 is of frequency 0.32 as the best method in co-registering T1 to PD synthetic brain scans, scale *κ* = 3 does not feature even once when registering 25 pairs of T2 and PD synthetic brain scans. Thus, compared with SSERg, MSERg has more accurate and robust registration performance across different datasets.

## Discussion

Co-registration of prostate *in vivo* MRI and *ex vivo* histopathology images is important in localizing prostate cancer extension on MRI and could help facilitate the development of radiomic machine based classifiers for characterization of prostate cancer on MRI^21, 22^. However, both the radiology and histopathology images undergo size and shape deformation during imaging and subsequent surgical resection and slice preparation, making automatic co-registration difficult. MSERg is an automatic registration method involving textural analysis scale selection, independent component analysis and spectral embedding to align the *in vivo* MRI and *ex vivo* histopathology images and thus map the ground truth tumor extension onto MRI.

Over the last decade there has been work on co-registration methods for pre-operative *in vivo* imaging and *ex vivo* histopathology images of the prostate. There are two general classes of methods to co-register *in vivo* MRI and *ex vivo* histopathology images of the prostate: 3D volume to volume registration^23, 24^ and 2D slice to slice registration^8–10^. 3D volume to volume registration requires for accurate 3D histology volume reconstruction^23, 24^. However, the 3D histology volume reconstruction is crippled by limited number of histology slices typically obtained during clinical workup of the surgical specimens^25^. Consequently, there has been several approaches that have attempted 2D slice to slice registration. The limitation of the 2D slice to slice registration is the requirement for the slice correspondence to first be determined between the *in vivo* MRI and *ex vivo* histopathology images. Xiao *et al*.^25^ developed an iterative group-wise comparison methods to identify the correspondence between *ex vivo* histology and pre-operative *in vivo* MRI slices. However, the accuracy of the correspondence estimation tends to depend on the manual segmentation of the prostate on the MRI sections, which in turn depends on the radiologist who annotates the tumor. Another recent attempt at histology-MRI co-registration involves the construction of 3D printed molds containing spatial landmarks to relate the histology slices with *in vivo* MRI slices^26^. While this approach could help address the constraints of establishing slice-to-slice correspondence, significant discrepancy might exist in the sagittal view between the 3D molds and MRI volume and it may not be possible to include this approach within a busy clinical protocol^26^.

Other approaches for co-registering MRI and pathology images have involved either some manipulation of the pathology specimens (e.g. inserting carbon rods^27^) or *ex vivo* imaging of the surgical specimen^23, 28, 29^. The closest work to our approach is that of Li *et al*.^6^. Li *et al*. applied ICA on multi-scale and multi-oriented Gabor features to fuse texture information from different image channels to drive multimodal co-registration of brain MRI scans. Their approach requires high quality images and landmark points to constrain the deformation field^6^. However,the prostate pathology and MRI scans such as the ones employed in our study may have various artifacts, making landmark selection subjective and user dependent. Ou *et al*.^7^ introduced a general-purpose multi-feature registration approach. They too employed Gabor filters and attempted to use feature selection approaches to identify the optimal feature attributes in order to increase the multimodal image similarity and voxel uniqueness within each monomodal image^7^. The authors did not explicitly attempt to evaluate possible correlation and hence redundancy between selected filters and limited the textural representations to only the Gabor filter family.

The underlying rationale behind MSERg, just like in Li *et al*.^6^ and Ou *et al*.^7^ is that alternative representations of multimodal images can reduce dissimilarity between multimodal imagery and help facilitate co-registration. The difference, however, between MSERg and the approaches of Li *et al*.^6^ and Ou *et al*.^7^ is that MSERg involves the use of spectral embedding on independent components derived from multiple classes of texture features (Haralick and Gabor) to further distill and rank the textural information to construct alternative representations of the original images. Figure 3 illustrates that spectral embedding representations appear to be more capable of improving the similarity between the fixed and moving images compared to the original intensity representation. Furthermore, MSERg aims to use the spectral embedding representations of different size scales to highlight different types of details that may be useful in identifying similarities between dissimilar images and thus improve registration performance. For example, small scale texture features from MRI and histopathology images may reflect capillary vessel infiltration, or small scale tissue differentiation, while large scale texture feature filters may detect larger blood vessels, bone, or boundaries between organs. MSERg specifically aims to explore the influence of length scale of textural representation, and thus the registration performance.

It has been shown that MSERg can improve the radiology and histopathology image co-registration performance. However, we do acknowledge that one weakness of this study, albeit a direction of future work, is the intelligent scale selection. Currently we used a fairly naïve scale selection approach for constructing MSERg. The approach essentially involves selecting scales based on their average single-scale registration performance on the independent learning set. However, this naïve scale selection method still gave us very promising and encouraging results. Clearly though more intelligent scale selection approaches can be employed. Future work could potentially employ optimization techniques for more efficient and intelligent scale combination selection. Another limitation of this work was that a precise and detailed sensitivity analysis of multiple different parameters involved in MSERg was not performed. For instance, we did not evaluate the potential variations in performance as a function of the number of features for ICA, the number of ICs for SE and the number of SE vectors for each SE representation. This is another direction for future work. Finally, though the learning and testing sets used in this study were from two different institutions, more validation experiments need to be performed on multiple datasets from different institutions in order to more thoroughly evaluate the robustness of MSERg.

## Data Description and Experimental Design

### Dataset Description

This study consists of both a clinical as well as a synthetic dataset. In the clinical dataset, we considered 45 pairs of *ex vivo* prostate histology specimens and corresponding 3 Tesla T2-w axial *in vivo* MRI from a total of 19 patients. Histology specimens were acquired after radical prostatectomy and sliced at 3 mm intervals. Each slice was stained with Hematoxylin and Eosin. The histology images were originally collected in quadrants and stitched into pseudo whole mount slices using Histostitcher © software^1^. All data was analyzed retrospectively, after de-identification of all patient sensitive information. All experimental protocols were approved under the IRB protocol #02–13–42*C* with the University Hospitals of Cleveland Institutional Review Board, and all experiments were carried out in accordance with approved guidelines. Under this IRB, we were allowed to obtain de-dentified images from St Vincent’s Hospital and University of Pennsylvania, and material transfer agreements were signed and agreed upon between Case Western Reserve University and University of Pennsylvania and St. Vincent’s Hospital. Correspondences between histology and MRI and cancer region annotations on the histology image and MRI were made by a pathologist and a radiologist working together in unison. Here, we treated the MRI as the fixed image and corresponding histology image as the moving image.

The synthetic dataset contains 50 corresponding slices of 181 × 217 PD, T1 weighted and T2 weighted brain MRI from the MNI BrainWeb simulated brain database^30^. The slice thickness of the dataset was 1 mm and intensity non-uniformity was 0%. We applied synthetic non-linear B-splines deformations on the PD images and then registered the corresponding T1 weighted and T2 weighted images in order to recover the original applied deformation. We then compared the recovered deformation against the induced deformation to evaluate performance of the co-registration scheme.

### Pre-processing of MRI and Pathology Data

The prostate regions of T2-w MRI slices were manually segmented using 3D Slicer^31^. The RGB digital images were converted to grayscale using MATLAB rgb2gray function, padded and down sampled to about 10 *μm* pixel resolution to match the resolution of the reference MRI. Each pair of pre-processed MRI and histology images were spatially concatenated to make sure that the same ICs were extracted on both the MRI and histology images. Texture feature extraction, ICA and SE were applied on the concatenated images.

### Experimental Design

#### Single-scale Representation Registration

In our study, we constructed new SE representations for 8 different scale *κ* to concentrate texture information with 8 different sizes of neighborhood. The normalized mutual information (NMI) was treated as an evaluation measure to evaluate the improvement in similarity of SE representations compared against the original MRI and the signal intensity from the histology slices alone. This single-scale spectral embedding representation based registration employed single-scale SE representations for the moving and fixed images and was performed on 20 pairs of MRI and histology images and 25 pairs of synthetic images in the learning set and then the registration results were evaluated in terms of DSC for clinical data and MDD for synthetic data at each *κ* ∈ {3, 5, 7, 9, 11, 13, 15, 17}. The top 3 performing scale representations were selected for MSERg based on the average registration performance in the learning set.

#### Multi-scale registration

The top 3 individual scale representations identified in the learning set were combined in a high dimensional registration space (Figure 2). MSERg employs *α*-MI as the similarity measure to conduct high dimensional registration on both the clinical and synthetic testing sets. The goal of this experiment was to evaluate whether combining the top performing individual scale representations can further improve multi-modal registration performance. In this work we compared MSERg with four other representation-based registration strategies, intensity-based registration^16^, texture-feature-based registration^9^, SIFT flow registration^19^ and single-scale representation based registration.

#### Intensity-based registration, texture-feature-based registration and SIFT flow registration

Here, ‘intensity-based registration’ refers to registration using the original intensity images as the fixed and moving images with MI estimated on the basis of a histogram as the similarity measure. The registration parameter selection and optimization were done during the learning process and on the training step. The set of parameters that resulted in the best registration result were locked down for the evaluation on the hold out testing set. For the texture-feature-based registration, we adopt MACMI method to selected the texture feature representations. MACMI selects the texture features that maximize the combined mutual information (CMI)^8^. We extracted an ensemble of five features with optimal CMI based on the same gradient, first and second order statistical features used in the work of Chappelow *et al*.^8^. Following feature selection, free form deformed registration^32^ was applied with *α*-MI as the similarity measure to co-register the multi-modal images in the testing set. In addition, we have validated with SIFT flow registration as proposed in the work of Liu *et al*.^19^.

#### Ground Truth Registration

In order to quantitatively evaluate the performance of the different co-registration methods in the clinical dataset, we need a ground truth definition of what constitutes a near optimal registration. This can then be used as the framework for comparing all other registration results. Towards this end, a control-point-based manual registration scheme was employed for co-registering all 45 pairs of histology-MRI sections. The corresponding control points were carefully selected by a pathologist and a radiologist on *ex vivo* histology images and *in vivo* MRI. The landmarks used for each registration pair varied between 6 to 10. The registration results were then visually assessed by the radiologist and pathologist. If they were found to be unsatisfactory, the registration was re-done. Once the co-registrations were found to be satisfactory, they were designated as the “ground truth” registration for the particular MRI-pathology pair.

#### Performance Evaluation Measures

Registration results were assessed via DSC^33^ and RMSD. DSC is defined as follows,

1 $$DSC({X}_{0},{X}_{1})=\frac{\mathrm{2|}{A}_{{X}_{0}}\cap {A}_{{X}_{1}}|}{|{A}_{{X}_{0}}|+|{A}_{{X}_{1}}|},$$

where ${A}_{{X}_{0}}$ denotes the region of interest (ROI) previously manually delineated on the fixed images. Here ROI refers to the capsule segmentation in global accuracy evaluation and the tumor annotated regions on the fixed images in local accuracy evaluation. *X*_0_ and *X*_1_ refer to the fixed and moving images respectively. The tumor annotations on the fixed image are obtained via manual registration between the histopathology images and MRI. ${A}_{{X}_{1}}$ refers to corresponding annotations on the moving images.

RMSD is defined as follows,

2 $$RMSD({X}_{0},{X}_{1})=\sqrt{\frac{1}{K}\sum _{k}^{K}{\Vert {X}_{0}({\beta }_{k})-{X}_{1}({\beta }_{k})\Vert }_{2}^{2}},$$

where *β*_*k*_ denotes the pixel of kth landmark in *X*_0_ and *X*_1_ and $\Vert \cdot \Vert$ represents the *L*_2_ norm. RMSD measures the distance between the anatomical landmarks such as the urethra, and nodules annotated by the pathologist and radiologist.

The MDD refers to

3 $$MDD({D}_{0},{D}_{1})=\frac{\sum _{c}^{C}({D}_{0}-{D}_{1})}{C},$$

where *D*_0_ is the ground true deformation field and *D*_1_ is the result deformation field. C is the total number of deformation vectors in the deformation field. MDD measures the difference between the registration result and the ground truth.

### Implementation Details

#### SE representations

To construct SE representations for different length scales, firstly, Gabor and Haralick features were extracted for *κ* ∈ {3, 5, 7, 9, 11, 13, 18, 17}. Then ICA was employed on the Gabor and Haralick features across different *κ* using the JADE-ICA algorithm. Finally, the spectral embedding algorithm was used to get the top 3 SE vectors for each individual scale representation.

#### Identifying optimal scales

In the learning set, single-scale SERg co-registration was implemented within each individual scale SE space. *α*-MI was applied as the high dimensional similarity measure with *k* - NNG as the entropic graph. The deformable registration was realized via the Elastix toolbox^34^ with B-spline interpolation. We set *α* = 0.99, *k* = 20 nearest neighbors with 3 resolution levels (resolution scales *η* = 4.0, 2.0, 1.0) employed with 100 iterations for each resolution level. The top 3 representations with the greatest DSC were selected as the optimal scale representations for MSERg.

#### Comparing MSERg against other representation-based registration methods

All the registration methods adopted the same standard gradient descent optimization method via the Elastix toolbox^34^. MSERg was compared against intensity-based registration and texture-feature-based registration. For Intensity-based registration, 3 resolution levels (resolution scales *η* = 4.0, 2.0, 1.0) were employed with 1000 iterations for each resolution level. The texture-feature-based registration also were made to use the same multi-resolution registration strategy with 100 iterations for each resolution level. MSERg employed a combination of 3 scale SE representations (*κ* ∈ {5, 7, 17}), with *α* = 0.99, *k* = 20 for *α*-MI registration in Elastix and 100 iterations per resolution level (resolution scales *η* = 4.0, 2.0, 1.0). SIFT flow was implemented via the Image Alignment Toolbox (IAT), which is a Matlab toolbox for image alignment and registration^35^. The manual registration to obtain the ground truth for evaluation of the different schemes was implemented via an in-house software tool using thin-plate splines^36^ method.

#### Computational efficiency

For a pair of MRI and histology images with 320 × 320 resolution, the spectral embedding representation construction for all 8 scales takes around 10 minutes and the representation construction for MACMI and SIFT takes 5 minutes and 7 seconds respectively. In the registration step, each single-scale spectral embedding representation registration requires around 1 minute to align a pair of MRI and histology images. Thus, it takes 1 × 8 × 20 = 160 minutes for all 8 single-scale representations in the learning set consisting of 20 cases. This computational burden can be greatly reduced via parallel computing because all the single-scale representation registrations are independent of each other. In the testing set, MSERg, MACMI and intensity-based registration take around 2.5, 3.5 minutes and 0.5 minute respectively. All three registration methods were implemented in the Elastix toolbox^34^ on a computer with a Windows 10 operation system based off a 3.40 GHz processor with 16.0 GB RAM. SIFT registration was implemented via SIFT flow^19^ algorithm taking about 1.5 minutes for each case on the same computer, using the same software and OS configuration. Table 2 summarizes the computational efficiency for all registration methods used in this paper.

**Table 2:** Computational efficiency including the image representation calculation and registration time consumption.

Registration methods	Intensity-based	MACMI	MSERg	SIFT
Representation computation	NA	5 min	10 min	7 sec
Registration	0.5 min	3.5 min	160 min for learning; 2.5 min for testing	1.5 min

Although MSERg has a higher computational cost during the learning step, this burden can be greatly reduced via parallel computing because all the single-scale representation registrations are essentially independent of each other, with no dependencies.

## Electronic supplementary material

Supplementary information
