# Supplementary material for: Co-Registration of ex vivo Surgical Histopathology and in vivo T2 weighted MRI of the Prostate via multi-scale spectral embedding representation
Source: Sci Rep. 2017 Aug 18;7:8717. doi: 10.1038/s41598-017-08969-w (PMC5562695; doi:10.1038/s41598-017-08969-w)
Supplement: Supplementary file 1 — Supplementary information [file 41598_2017_8969_MOESM1_ESM.pdf]

Co-Registration of *ex vivo* Surgical Histopathology and *in vivo* T2 weighted MRI of the Prostate via multi-scale spectral embedding representation

Lin Li[1,*], Shivani Pahwa[2], Gregory Penzias[1], Mirabela Rusu[3], Jay Gollamudi[4], Satish Viswanath[1], and Anant Madabhushi[1,]**

[1]Department of Biomedical Engineering, Case Western Reserve University, Cleveland, Ohio, United States of America, 44106
[2]Department of Radiology, Case Western Reserve University, Cleveland, Ohio, United States of America, 44106
[3]GE Global Research, Niskayuna, New York, United States of America, 12309
[4]University Hospitals, Cleveland, Ohio, United States of America, 44106
[*]Corresponding Author (e-mail: lxl477@case.edu)
[**]Contact Author (e-mail: axm788@case.edu)

Supplementary Information

We are glad to public the MSERg code for testing by others. This code can be located at https://github.com/lynli/MSERg.

Methods

Notation

Let X_0 be the fixed image and X_1 be the moving image. X_T represents the transformed moving image $X_T = T(X_1)$, where T refers to the transformation applied to X_1. $X_0 = (C, f)$ in which C is an n dimensional grid image scene and $f(c)$ is the intensity value at location $c \in C$. $f^t_{j,\kappa}(c)$ represents the corresponding texture values at $c \in C$ after applying a textural operation on the image scene C and where $j \in \{1,2,3....,P\}$ represents the jth texture features extracted and κ is the corresponding filter length scale size, $\kappa \in \{3,5,7,9,11,13,15,17\}$. $f^{ICA}_{m,\kappa}(c)$, where $m \in \{1,2,....M\}$, represents the mth ICA vector derived from the texture features extracted at scale κ. $f^{SE}_{s,\kappa}(c)$, where $s \in \{1,2,....S\}$, represents the sth spectral embedding vector based on the ICs extracted at scale κ. More specific notational descriptions are shown in **Table** 1.

SE representation Construction

MSERg comprises two main modules. The first module involves constructing representations of the SE spaces and the second module involves multi-scale registration using the SE vectors. The module on SE representation construction involves feature extraction, ICA and SE at each individual length scale. (see Table 2)

Feature Extraction

The procedure involves spatially concatenating X_0 and X_1. Let $F^t_\kappa(c) = [f^t_{1,\kappa}(c), f^t_{2,\kappa}(c), \ldots, f^t_{P,\kappa}(c)]$ where $f^t_{1,\kappa}, f^t_{2,\kappa}, \ldots, f^t_{P,\kappa}$ comprise a set of P features at scale κ from the concatenated X_0 and X_1. Our features include Gabor filters[1] with 10 orientations ($\theta = \frac{i\pi}{10}, i \in \{1,2,...10\}$) and 13 Haralick features[2] for each scale $\kappa \in \{3,5,7,9,11,13,15,17\}$. Gabor features are multi-scale steerable filters which capture the gradient responses across multiple different orientations[3] and Haralick features extract the information about the co-occurrence of signal intensities within the image[4]. These two classes of texture features are thus able to provide alternative image representations for multi-modal co-registration.

Independent Component Analysis

Let $F^{ICA}_\kappa(c) = [f^{ICA}_{1,\kappa}(c), f^{ICA}_{2,\kappa}(c), \ldots, f^{ICA}_{M,\kappa}(c)]$ where $f^{ICA}_{1,\kappa}, f^{ICA}_{2,\kappa}, \ldots, f^{ICA}_{M,\kappa}$ denote a set of M independent components of each scale κ. Our approach uses the JADE-ICA algorithm[5,6] to extract ICs from the Gabor and Haralick features at each scale κ. X_0 and X_1 have already been spatially concatenated, so texture feature extraction and ICA are performed on both X_0 and X_1 simultaneously, in turn, ensuring that the F^{ICA}_κ is linked to X_0 and X_1.

Spectral Embedding

The aim of SE is to project data from M dimensions to S dimensions, where $S < M$. This creates a non-linear embedding from which the first S most important features can be identified for subsequent use in α-MI based multi-modal co-registration. SE allows the content of the final S components to be optimized by finding a mixture of all the M ICs. This yields $F^{SE}_\kappa(c) = [f^{SE}_{1,\kappa}(c), f^{SE}_{2,\kappa}(c), \ldots, f^{SE}_{S,\kappa}(c)]$. Note that $f^{SE}_{1,\kappa}, f^{SE}_{2,\kappa}, \ldots, f^{SE}_{S,\kappa}$ denote a set of S SE vectors at scale κ. The SE of scale κ, F^{SE}_κ, can be obtained by solving,

$$F^{SE}_\kappa = \arg\, min \left(\frac{\sum_l \sum_r \left\| F^{SE}_\kappa(r) - F^{SE}_\kappa(l) \right\| \omega_{rl}}{\sum_r F^{SE}_\kappa(r)^2 d(r)} \right), \tag{1}$$

where ω_{rl}, the edge weights between pairwise observations r and l. Each location (r,l) within the weight matrix $W = [\omega_{rl}] \in R^{|C|\times|C|}$, r, $l \in C$, where $\omega_{rl} = exp(\frac{-\left\| f^{ICA}_{r,\kappa} - f^{ICA}_{l,\kappa} \right\|_2^2}{\delta^2})$. Here δ is a scale parameter, and $d(r) = \sum_l \omega_{rl}$. The minimization of Equation (1) reduces this to an eigenvalue decomposition problem,

$$(D - W)F^{SE}_\kappa = \lambda D F^{SE}_\kappa, \tag{2}$$

where D is a diagonal matrix, $D_{rr} = \sum_l W_{rl}$ and λ represents the eigenvalues. In this work we decided to choose the top 3 embedding vectors from SE for each scale κ. Hence $F^{SE}_\kappa(c) = [f^{SE}_{1,\kappa}(c), f^{SE}_{2,\kappa}(c), f^{SE}_{3,\kappa}(c)]$, where $c \in C$, associated with the smallest eigenvalues λ_1, λ_2, λ_3 are selected as the new image representations to be used during registration step. Thus each individual scale SE representation consists of 3 SE vectors for both the moving and the fixed images.

Table 1. Symbols Description.

Symbols	Description	Symbols	Description
X_0	fixed image	X_1	moving image
Ω_0	fixed image field	Ω_1	moving image field
X_T	transformed moving image	C	image scene
c	image grids in C	κ	scale index
$\mathscr{M}_0$	ROI annotation mask of X_0	$\mathscr{M}_1$	ROI annotation mask of X_1
$\mathscr{M}_T$	transformed ROI annotation mask of X_1	f	intensity representation
f^t	textural representation	F^t	set of textural representations
f^{ICA}	ICA representation	F^{ICA}	set of ICA representations
f^{SE}	SE representation	F^{SE}	set of SE representations
j	texture feature index	m	ICs index
s	SE vector index	θ	orientation in Gabor feature
W	weight matrix	ω	elements of weight matrix W
r,l	coordinate locations in W	$d(r)$	sum of weight elements at location r
μ	transformation parameters	ζ	registration cost function
T	transformation matrix	Γ	sum of k nearest neighborhood distance
Ψ	registration dimensions	α	an α-MI constant
q	number of scales used in MSERg	p	the index of the nearest neighborhood pixel
γ	an α-MI constant	A	region of interest in DSC computation
ρ	number of pixels of $X_0 \cap X_1$	E	entropy
β	the pixel index of landmark on the images	k	the landmark index

Table 2. Algorithm 1: SE Representation.

Input: $[X_0, X_1], \kappa$
Output: $[F^{SE}_{X_0,\kappa}, F^{SE}_{X_1,\kappa}]$
For all scale κ **do**
$[F^t_{X_0,\kappa}, F^t_{X_1,\kappa}] = Texture([X_0, X_1], \kappa)$;
$[F^{ICA}_{X_0,\kappa}, F^{ICA}_{X_1,\kappa}] = ICA([F^t_{X_0,\kappa}, F^t_{X_1,\kappa}])$;
For i = 1:number of ICs **do**
$M(:,i) = vectorize([F^{ICA(i)}_{X_0,\kappa}, F^{ICA(i)}_{X_1,\kappa}])$;
Endfor;
Calculate distance matrix W of M;
$[F^{SE}_{X_0,\kappa}, F^{SE}_{X_1,\kappa}] = \arg min \left(\frac{\sum_l \sum_r \left\| F^{SE}_\kappa(r) - F^{SE}_\kappa(l) \right\| \omega_{rl}}{\sum_r F^{SE}_\kappa(r)^2 d(r)} \right)$;
w_{rl} is the element of W at location (r,l) and $F^{SE}_\kappa = [F^{SE}_{X_0,\kappa}, F^{SE}_{X_1,\kappa}]$.
Endfor

Table 3. Algorithm 2: Multi-scale registration.

**Input:** $F^{SE}_{X_0,\kappa}, F^{SE}_{X_1,\kappa}, X_0, X_1, \mathscr{M}_0, \mathscr{M}_1$
**Output:** $\mathscr{M}_T, X_T$
**For** all scale κ **do**
$\mu = \arg min_\mu \alpha MI(T_\mu : F^{SE}_{X_1,\kappa} \to F^{SE}_{X_0,\kappa})$;
$\mathscr{M}_{T_\kappa} = T_\mu(\mathscr{M}_1)$;
$X_{T_\kappa} = T_\mu(X_1)$;
$CR_\kappa = CR(X_0, X_{T_\kappa})$;
$DSC_\kappa = DSC(\mathscr{M}_0, \mathscr{M}_{T_\kappa})$;
**Endfor**
Select the best performing individual scales l, n, p :
$l, n, p = \arg max(CR_\kappa, DSC_\kappa)$;
Fixed representation $F_{fix} = [F^{SE}_{X_0,l}, F^{SE}_{X_0,n}, F^{SE}_{X_0,p}]$
Moving representation $F_{move} = [F^{SE}_{X_1,l}, F^{SE}_{X_1,n}, F^{SE}_{X_1,p}]$;
$\mu = \arg min_\mu \alpha MI(T_\mu : F_{move} \to F_{fix})$;
$\mathscr{M}_T = T_\mu(\mathscr{M}_1)$; $X_T = T_\mu(X_1)$;

Multi-scale Registration

Optimal Scale Selection

On the learning set, we conducted single-scale SERg which means that the transformation of moving image X_T is driven by SE vectors obtained over texture features extracted at a single fixed scale. The top 3 individual scales were identified in terms of DSC and CR measures on the learning set. In this work, we limited our new representation spaces to 3-scale combinations, though the approach could be extended to invoking more than 3 scales as well. We stack the 3 SE vectors and employed the vectors for α-MI based registration. Once the optimal scales and representations were identified on the learning set, the same set of scales and representations are then used via MSERg for the cases on the testing set. (see Table 3)

α-MI Registration

In MSERg, we performed Ψ-dimensional registration with $\Psi = 3q$, where q denotes the number of scales used in multi-scale registration. The registration process involves alignment of a Ψ-dimension moving image $X_1 : \Omega_1 \subset R^\Psi \to R$ to a fixed image $X_0 : \Omega_0 \subset R^\Psi \to R$ can be formulated as an optimization problem:

$$\widehat{\mu} = \arg min_\mu \zeta(T_\mu : \Omega_1 \to \Omega_0), \tag{3}$$

with μ being the vector of transformation parameters $T_\mu : \Omega_1 \to \Omega_0$ and ζ being a suitable cost function (here α-MI). Let $F^{SE}_{X_0,\kappa}(c)$ be the fixed image feature vector in image at location c of scale κ and $F^{SE}_{X_T,\kappa}(c)$ be the corresponding moving feature vector at location c of scale κ. Let $F^{SE}_\kappa(c) = [F^{SE}_{X_0,\kappa}(c), F^{SE}_{X_T,\kappa}(c)]$ be the concatenation of the two feature vectors. To eliminate the limitation of histogram entropy estimation, we applied the graph-based entropy estimation by constructing three kNNG in $F^{SE}_{X_0,\kappa}$, $F^{SE}_{X_T,\kappa}$ and F^{SE}_κ. The total Euclidean distance of a feature vector to its k nearest neighbors can be calculated as described below:

$$\Gamma^0_c = \sum_p^k \left\| F^{SE}_{X_0,\kappa}(c) - F^{SE}_{X_0,\kappa}(c_p) \right\|, \tag{4}$$

$$\Gamma^T_c = \sum_p^k \left\| F^{SE}_{X_T,\kappa}(c) - F^{SE}_{X_T,\kappa}(c_p) \right\|, \tag{5}$$

$$\Gamma^{0T}_c = \sum_p^k \left\| F^{SE}_\kappa(c) - F^{SE}_\kappa(c_p) \right\|, \tag{6}$$

where c_p denotes the location of pth nearest neighbor within kNNG of location $c \in C$, where $p \in \{1,2,3,\ldots,k\}$. So a graph-based mutual information can be formulated as following:

$$\alpha MI(\mu; F^{SE}_{X_0,\kappa}, F^{SE}_{X_T,\kappa}, F^{SE}_\kappa) = \frac{1}{\alpha - 1} \log\left(\frac{1}{|C|^\alpha} \sum_{c=1}^{|C|} \left(\frac{\Gamma^{0T}_c}{\sqrt{\Gamma^0_c \Gamma^T_c}}\right)^{2\gamma}\right), \tag{7}$$

with $\gamma = \Psi(1-\alpha)$ and $\alpha \in (0,1)$.

Multi-attribute combined mutual information (MACMI) registration

MACMI selects the texture features that maximize the combined mutual information (CMI)[7]. CMI is calculated by equation (8). $F^{t_1}F^{t_2}\ldots F^{t_n}$ represents the texture feature ensemble and E denotes the entropy.

$$CMI(X_0, X_t F^{t_1}F^{t_2}\ldots F^{t_n}) = E(X_0) + E(X_t F^{t_1}F^{t_2}\ldots F^{t_n}) - E(X_0 X_t F^{t_1}F^{t_2}\ldots F^{t_n}) \quad (8)$$

The features that maximize CMI and hence the $E(X_t F^{t_1}F^{t_2}\ldots F^{t_n})$ are selected. In our implementation of MACMI, we selected five features because ensembles of more than five features will result in sparse gray level histograms, making it near impossible to accurately estimate the joint entropy.

References

1. Daugman, J. G. Complete discrete 2-d gabor transforms by neural networks for image analysis and compression. *Acoust. Speech Signal Process. IEEE Transactions on* **36**, 1169–1179 (1988).

2. Miyamoto, E. & Merryman, T. Fast calculation of haralick texture features. *Hum. computer interaction institute, Carnegie Mellon Univ. Pittsburgh, USA. Jpn. restaurant office* (2005).

3. Narani, B. & Shenbagavalli, A. Image registration by feature based information fusion. In *IJCA Proceedings on International Conference in Recent trends in Computational Methods, Communication and Controls (ICON3C 2012)*, 2 (Foundation of Computer Science (FCS), 2012).

4. Haralick, R. M., Shanmugam, K. & Dinstein, I. H. Textural features for image classification. *Syst. Man Cybern. IEEE Transactions on* 610–621 (1973).

5. Cardoso, J.-F. & Souloumiac, A. Blind beamforming for non-gaussian signals. In *IEE Proceedings F-Radar and Signal Processing*, vol. 140, 362–370 (IET, 1993).

6. Cardoso, J.-F. High-order contrasts for independent component analysis. *Neural computation* **11**, 157–192 (1999).

7. Chappelow, J., Madabhushi, A., Rosen, M., Tomaszeweski, J. & Feldman, M. A combined feature ensemble based mutual information scheme for robust inter-modal, inter-protocol image registration. In *Biomedical Imaging: From Nano to Macro, 2007. ISBI 2007. 4th IEEE International Symposium on*, 644–647 (IEEE, 2007).
